# Book Review

**Published:** 1908-09

**Authors:** 


**Les venins des Animaux Venimeuse et la Sérothérapie Anti-venimeuse.** By A. CALMETTE. Paris: Masson. 1907.—In this volume Calmette has gathered together his publications and observations of the last fifteen years. It forms an admirable record of patient work. Those interested in the subject will find it of the utmost value for reference. After detailing the morphology of the snakes of Europe, Asia, Africa, Australia and America, the author discusses the physiological actions of the several venoms, and the value of vaccination and anti-toxin treatment. The poisons of certain fishes, batrachians, saurians, and mammals are then considered. We must express our appreciation of the numerous illustrations. These alone make the book worth its money.

**The Bacteriological Examination of Disinfectants.** By WILLIAM PARTRIDGE, F.I.C. Pp. 66. London: The Sanitary Publishing Co. 1907.—This brochure, which is introduced by a preface by Major C. E. P. Fowler, will be welcomed by those who have to deal with matters of practical sanitation. The difficult problems met with in determining the values of the various disinfectants are fully discussed, and the technique of the methods given in detail. There is a special chapter on the influence of organic matter on disinfectants.

**The Reduction of Cancer.** By the Hon. ROLLO RUSSELL. Pp. 62. London: Longmans, Green & Co. 1907.—This strange little sermon appearing in scientific guise, although pleasantly enough written and insisting on a sound mode of living, has little or no real claim to its title or to a place in scientific literature. The burden of its teaching may be gathered perhaps from a single sentence: "All ingesta not really nutritious, and containing a strong poison which pleasantly excites, are as a matter of fact, when habitually and largely consumed, not only luxuries, but destructive luxuries." From this text, by a careful compilation of statistical extracts, almost any proposition might be bolstered up, and the remainder of the book goes to show that "in a long list of countries *all* the small consumers of flesh, tea, coffee, beer and tobacco have a small cancer rate; *all* the large consumers have a high rate." But the author's arguments are very loosely strung together. Many authorities are laid under contribution, general non-committal statements are strained into direct proofs; dietetic precautions in cases of aneurysm are represented as "having a bearing on liability to cancer" which "is not remote," until the brain reels under the battery of eminent names, sensational figures, and hasty generalisation into a sort of submissive condition in which one would readily admit that Tariff Reform is the cause of tuberculous meningitis, and a Radical Government responsible for sporadic cretinism. Still, cancer apart, there is much good advice in Mr. Russell's book, and if only its title attracts attention to his teaching (which the Greek philosopher could sum up in two words, Μηδὲν ἄγαν), there will be no reason to complain of the unscientific reasoning in its pages.

**The Opsonic Method of Treatment.** By R. W. ALLEN, M.B., B.S. (Lond.). Pp. ix., 138. London: H. K. Lewis. 1907.—This small work gives us a brief but clear *résumé* of the chief facts of the opsonic method. It cannot fail to be of interest, coming as it does at a time when many medical men are still sceptical of the value of the opsonic index, more especially in its relation to the diagnosis of the various tuberculous infections. The chapter on technique is excellent, but though much stress is laid on the necessity for good technique, very little is said about the errors which appear to us to be inseparable from the estimation of the opsonic index, even in the hands of the most capable and conscientious workers. The writer deals briefly with the various infections amenable to serum therapy, and indicates the chief points in the preparation and administration of suitable vaccines. Some of the sections appear to us to be too short, notably that dealing with infections due to the Bacillus coli communis. On the whole, the book is compact and interesting, if a trifle optimistic.

**Skin Affections in Childhood.** By H. G. ADAMSON, M.D. Pp. xvi., 287. London: Henry Frowde and Hodder & Stoughton. 1907.—A welcome attempt is made in this handbook to depart from the trammels of a vile nomenclature which has in every direction rendered dermatology a closed path to students, whether qualified or unqualified, and there can be no doubt that the causal classification of skin diseases places the subject at once on a fresh footing, where the scientific practitioner can fairly hold his own with the charlatan who once roamed fancy free over the integuments of suffering mankind. Dr. Adamson classifies the skin affections of children under: Affections of congenital origin; eruptions due to local physical causes; eruptions due to animal parasites; eruptions due to vegetable parasites; eruptions due to local microbic infection; affections probably of local microbic origin; tuberculosis of the skin; eruptions of toxic origin, or the result of general microbic infection; affections of nervous origin; unclassified affections. And it is surprising how small the last class comes to be, including, as it does, eczema, psoriasis, lichen, pityriasis rosea, prurigo of Hebra, urticaria pigmentosa, and alopecia, illustrating, if proof were needed, how far the science of dermatology has advanced since the "dermatographic survey" to which Willan subjected skin diseases. The result is a short, accurate description of many troublesome affections, whose pathology, where understood, is excellently set out, with rational hints as to treatment, and a total absence of the imaginative causative ingenuity with which outraged nature has been so abundantly credited in many text-books of dermatology in the past. Dr. Adamson is a careful observer, not only of children's diseases, but of children, and his book is worth reading.

**A Guide to the Administration of Ethyl Chloride.** By G. A. H. Barton, M.D. Second edition. Pp. 54. London: H. K. Lewis. 1907.—In the second edition of his monograph on the administration of ethyl chloride Dr. Barton has introduced some useful additions to the information contained in the first edition. The dosage is very practically given, and a suggestion is made of a method for regulating the period of anæsthesia by the withdrawal of the face piece at definite and clearly indicated stages of reflex phenomena. This book will probably be the means of preventing some accidents with ethyl chloride, and is therefore a useful work.

**A Text-Book of Mental and Sick Nursing.** By Robert Jones. M.D., B.S. Pp. xix., 222. London: The Scientific Press Limited. 1907.—The book opens with a plea for a combination of medical and surgical together with mental training, but it does not suggest how such medical and surgical education is to be acquired by the male nurse, a difficulty the solution of which is not very obvious. The opening chapters give a short account of the physiology of the various organs — the brain, the circulatory and respiratory system, the kidneys and the organs of digestion. The section relating to the mind is written in a very clear style, and explains step by step the aberrations from the normal of a deranged mind, and by describing the connection which exists between the impressions received from without, and our thoughts and actions, it illustrates very simply the terms "hallucination," "illusion" and "delusion;" it draws, in fact, a neat picture of the difference between sanity on the one hand and insanity on the other. Chapters X. to XIII. deal with the duties of a mental nurse, and are calculated to embue those who read them with an increased sense of responsibility, and to impress upon the nurse's mind the importance of noticing and reporting to the medical man in charge all the minute variations in the mental aspect of the case. A good account is also given of the necessary precautions to be taken with regard to the patient's surroundings. The latter part of the book is devoted to a description of the technique of nursing, especially as applied to the different phases of mental diseases. A difficult task has been tackled in a masterly manner, and a long-felt want has been met by the clever way in which Dr. R. Jones has brought theory into line with practice by this carefully-written manual, which contains many suggestions for those responsible for the training of mental nurses, as well as a fund of information for the latter.

